# The duty to bring children living in conflict zones to a safe haven

**DOI:** 10.1080/17449626.2016.1247744

**Published:** 2016-12-14

**Authors:** Gottfried Schweiger

**Affiliations:** ^a^ Centre for Ethics and Poverty Research, University of Salzburg, Salzburg, Austria

**Keywords:** Children’s rights, human rights, children in conflict zones, child refugees, human rights intervention

## Abstract

In this paper, I will discuss a children’s rights-based argument for the duty of states, as a joint effort, to establish an effective program to help bring children out of conflict zones, such as parts of Syria, and to a safe haven. Children are among the most vulnerable subjects in violent conflicts who suffer greatly and have their human rights brutally violated as a consequence. Furthermore, children are also a group whose capacities to protect themselves are very limited, while their chance to flee is most often only slim. I will then discuss three counterarguments: the first counterargument would be that, instead of getting the children out of a particular country, it would be better to improve their situation in their home countries. A second counterargument could be that those states, which have such a duty to bring children to a safe haven, would be overburdened by it. Finally, the third counterargument I want to discuss states that such a duty would also demand a military intervention, which could worsen the situation even further.

In this paper, I will discuss a children’s rights-based argument for the duty of states, as a joint effort, to establish an effective program to help bring children out of conflict zones, as is the case in Syria at present. My argument will be presented in three steps: firstly, children are among the most vulnerable subjects in violent conflicts and that they suffer greatly as a consequence. I will employ a children’s rights framework to support this claim. Furthermore, children are also the group whose capacities to protect themselves are very limited, while their chance to flee is most often only slim (and requires others helping them). As such, children are a group that, on the one hand, deserve special attention, and, on the other hand, are not able to move out of danger on their own. This seems to provide a sufficient basis to establish a duty to be proactive and bring children to safe countries. Children also have a right to a family and it is proven that staying with their caregivers, to whom they have close attachment, is critical; as such, this duty should also include caregivers and other close family members. I will then, thirdly, discuss three counterarguments: the first counterargument would be that, instead of getting the children out of the country in conflict, it would be better to improve their situation in their home countries. While I agree that this would indeed be a better outcome, I will argue that this is not very realistic, and in the meantime, we should bring children out of conflict zones, possibly with the option to return them as soon as the situation sufficiently improves. A second counterargument could be that those states, which have such a duty to bring children to a safe haven, would be overburdened by it. I will contend that the duty I propose is limited by external factors but that there are good reasons to believe that the highly developed countries are able to fulfill it without being overburdened. Finally, the third counterargument I want to discuss states that such a duty would also demand a military intervention, which could worsen the situation. I will conclude that this danger exists, but that it can be limited. While bringing children out of conflict zones does not necessarily involve a high level of military engagement in these countries, it does not mean that sides need to be taken.

## 1. Children in conflict zones

For the purpose of this paper, I will assume that children do have rights and that the UN Convention of the Rights of the Child (CRC) is an adequate expression of these rights within the context of all human rights, but specified according to children’s particular needs and competences.^1^ I view these human rights of children as both moral and legal rights,^2^ while assuming that their reach overlaps in this regard. As moral rights, they are binding even if states choose to ignore them; meanwhile, as legal rights, they are established by international treaties. I am agnostic towards the question about whether children have such rights and how they can be justified philosophically (see, e.g. Archard 2004; Dixon and Nussbaum 2012). The CRC sets out a number of rights derived from the overarching concept of children’s dignity, with what is in the best interests of children being of particular interest. For my purpose, it is not necessary to examine each and every right that children have under the CRC, nor how it is violated by living in conflict zones and the hardships that come with it. It is sufficient to point out that the situation is so bad that it clearly constitutes a human rights violation according to the CRC. The CRC states that each and every child has a right, for example, to health, decent living conditions, education, and protection from violence, abuse and neglect.

What is also important is that children’s rights under the CRC are universal, with each and every child entitled to the protection of their rights. This means that children’s human rights claims do not stop at national borders.^3^ This lies within the nature of human rights themselves and has moral and legal importance: from a moral point of view, we need to clarify what constitutes reasonable moral obligations, while, from a legal point of view, we need to ask what kinds of obligation can or do follow on from international human rights treaties, such as the CRC or other relevant laws. Although it has not been proposed as yet that the CRC provides the legal framework for a duty to bring children to a safe haven, I believe that both the moral and legal aspects of children’s human rights point in that direction. States and the UN, as well as other international organizations, already impose sanctions in response to human rights violations with a wide range of measures, including interventions and UN-led military operations. I am not concerned with the question about whom is responsible for a violent conflict and who should be punished for it. I am also highly skeptical that sanctions can actually improve the situation for children in conflict zones. It is rather the case that, in countries such as Syria or Iraq at this present time, those kinds of sanctions that are normally used to improve the situation regarding children’s rights have no conviction, It is pointless reminding the government of Syria that it is obliged to protect children’s rights because they do not have the means to do so effectively. Thus, the intervention from foreign states is permissible, and as I will argue later also obligatory, but in such a way that it maximizes the outcome for children with minimal risks and those who carry out the duty to bring them to a safe haven.

Unfortunately, we do not exactly how many children are currently living in conflict zones such as Syria and Iraq.^4^ There are estimations about how many children have arrived in the European Union (EU), while it can be reasonably expected that hundreds of thousands of children are currently living in the aforementioned conflict zones; meanwhile, globally, there are possibly millions of children affected. UNICEF estimated in February 2016 that about six million children are affected by the ongoing war in Syria and that nearly 2.5 million children are awaiting registration as refugees. UNICEF and other humanitarian organizations have reported gross violations of these children’s rights. Most of them – one report of the UN counts about two million for whom this is the situation right now – are out of school, and that this particular situation is a persistent issue, which might lead to a ‘lost generation’.^5^ In October 2015, UNICEF reported that they reached out to more than 430,000 children under five years of age, while pregnant and lactating women received multi-micronutrient supplementation, over 500,000 children aged between 6 and 59 months received nutrient supplements and more than 8000 children aged between 6 and 59 months were treated for global acute malnutrition.^6^ The toll on children’s minds and souls is maybe even harder to estimate, while it is without doubt that most will be traumatized and experience anxiety and fear in later life (one early study: Quosh, Eloul, and Ajlani 2013). Access to healthcare is also declining and the healthcare system is on the brink of collapse (in many areas, it already has collapsed due to a lack of drugs, electricity, etc.), which leaves millions of children without vaccinations or treatment in cases of illness or injury. Children suffer increasingly from diarrheal diseases, such as cholera, due to a lack of access to clean water, while the risk of pneumonia and other respiratory infections increases due to low temperatures during the winter, together with inadequate shelter, housing or heating. UNICEF reports that it has given winter kits to 152,000 children in the first three months of 2016 alone; 44,000 of those children are living in hard to reach areas. The long-term health effects of the conflict on children can, and unfortunately will, for many, be severe and irreversible (Devakumar et al. 2015). The rate of disabilities, mental and physical impairments, and chronic diseases will be especially high. The situation for all these children is bad, but for those trapped in conflict zones it is even worse:

The humanitarian consequences of the conflict in Syria has taken the toughest toll on communities, with children trapped in besieged locations where the most basic lifelines to the outside world have been cut off as a deliberate tactic of war. There are an estimated 486,000 people living in 18 locations designated as besieged with limited or no access to food, water, healthcare, electricity and fuel. UNICEF estimates half are children.^7^

Children in conflict zones are difficult to reach for humanitarian organizations; they are in acute danger of being injured or killed as well as often trapped in their situation. World Vision reported in March 2016 that about 12,000 children have been killed in Syria,^8^ while many more have been wounded. Since the outbreak of the conflict, over 250,000 people in total have been killed and over one million injured. It can only be estimated how many children have lost one or both of their parents, which leaves them even more vulnerable and in need of assistance and care.

The evidence I have presented in this section only gives a glimpse into the reality of these children’s lives in conflict zones. As the aid for them is severely underfunded and the conflict is ongoing, it cannot be expected that their plight will improve anytime soon. It is essential that children experience healthy development, care and protection, with their basic needs fulfilled; otherwise, they will suffer from long-lasting negative consequences. Violent conflicts, such as the war in Syria, undermine such needs beyond the direct and indirect consequences mentioned here, which makes these children more vulnerable to exploitation. There are some troublesome reports about children forced to work in Syria, being sexually abused – especially girls and young women – after they flee^9^ or even made to participate in the violent conflicts as child soldiers or human shields.

## 2. Children’s capacities to protect themselves and to flee

So far, I have shown that children are among the most vulnerable in conflict zones and that they suffer greatly under such circumstances. The hundreds of thousands of children actually living in places of civil war, such as Syria, are denied essential human rights, which they are granted under the CRC: namely, the right to life, health, education, safety, a decent living standard and so on. Although children represent a heterogeneous group, involving those who are completely dependent on others, as well as those who are on the border of adulthood, this claim about children’s vulnerability holds for them as a group.^10^ What is important now is that children are not only among the most vulnerable and the most affected by violent conflicts; they also only have a slim chance to escape and change their situation. This supports the second step in my argument concerning a duty to bring them out of conflict zones.

Children, then, obviously make up a very heterogeneous group, ranging from newborns to near adults until the age of 18 years. This makes it is somewhat more complicated to draw conclusions about children as a group, while it would go beyond the scope of this paper to discuss the concept of childhood in detail (in that respect, see, e.g. Schapiro 1999). Despite that, I want to claim that for all children, when we look at them as a group and not as individuals with differentiating features based on age or maturity, certain characteristics are crucial in terms of the proposed duty to bring them to a safe haven, which are also acknowledged in the CRC. Firstly, they are not fully developed physically and psychologically, which makes it harder, perhaps even impossible, for them to protect themselves from the dangers of violent conflicts and to uphold their human rights. For young children, this claim is uncontroversial since very young children cannot sustain themselves at all and may die without care and protection from others, while younger, preadolescent children are still at risk of being harmed and exploited by adults. Children cannot run a school on their own, nor can they provide themselves with medical care or rebuild their homes. In particular, if their parents are wounded, dead or captured, they are highly vulnerable. The case for older teenagers is certainly weaker in that regard, while some of them will even actively engage in violent behaviors and become combatants themselves, or otherwise take advantage of those who are younger and weaker.^11^ That said, those teenagers are not fully matured and still deserve protection. Joel Anderson and Rutger Claassen refer to a ‘regime of childhood’ (2012) without implying that children should not be held responsible for their actions.^12^ Such a regime does not focus on competences alone, but also on the wider implications of treating teenagers who are not fully mature as adults, even if, as I want to formulate it, this is in their best interest regarding the rights they have under the CRC. It is not, however, because it would certainly benefit the development of these children if they fell under the protection of the CRC, even if their vulnerability is not that different than that of adults. Furthermore, it is certainly the case that not all children are equally mature, while differential treatment would require a thorough analysis, which is not feasible under the circumstances of a conflict zone.^13^

Secondly, children’s limited capacities to flee and move to a safe haven themselves are equally limited. The pictures of a dead body lying on a Turkish beach were all over the news a few months ago and can serve as an example of the dangers of fleeing. Those dangers certainly exist for adults as well; but, for children, the dangers are simply higher because they are less prepared for the hardship that comes with fleeing, given that being alone on the run makes them more vulnerable to exploitation or being otherwise harmed by adults and because protection systems are weak, if they exist at all, under such circumstances (for more details, see Kanics et al. 2010). While younger children cannot flee alone, taking them along makes the journey more difficult for the family. It is also important to note that girls are more prone to certain forms of violence and abuse because of their gender. Fleeing alone is a traumatic experience in itself because children suffer from separation from their parents and are constantly facing experiences of loneliness and insecurity (Berman 2001). All these things considered, it is understandable why children are often trapped in conflict zones and only have very limited or no options to get to a safe haven on their own. At the same time, they cannot rely on the protection systems of the state because they are failing under such circumstances or because a law-free space has developed in the absence of authority.

## 3. The duty to bring children to a safe haven

I have shown that children’s rights are grossly violated in conflict zones, such as Syria, and that they are not safe there. I have further indicated that children lack the competences to protect themselves, while their opportunities to get to a safe haven on their own are very limited. Based on these two arguments, I will now flesh out a duty to bring children to safety and argue that this duty falls to those states which are able to fulfill it, not primarily as individual states but as a group under the heading of the UN. Furthermore, this duty also covers the concerned children’s families.

Let me begin by stating that the duty to protect children’s rights falls first and foremost to the state in which the children reside. This is a moral and a legal claim regarding the CRC, under which all states that signed it pledged to protect and promote children’s rights. The moral obligation is similar and I will not explore its basis here. Under some circumstances, states fail to do so, while, in cases of violent conflict and the collapse of state order, at least in some parts of a country, the protection of children’s rights becomes even more necessary, but at the same time more difficult, maybe even impossible, because the state in question no longer has the means to do so or simply lacks control over the space where children live. In such cases, a clear-cut attribution of the responsibility to protect children’s rights is impossible, although all individuals involved are required to respect those rights just as they have to respect human rights in general. Still, it is unclear who is responsible for providing children with adequate healthcare, education or shelter in cases where they are separated from their families. The reality of (civil) war and conflict often makes upholding such rights impossible. Even if little disagreement exists over the claim that violent conflicts actually violate children’s rights in several dimensions, it is less clear why other states may have a duty to protect these children and their rights, even under the scenario that the state, in which those children live, is unable (or unwilling) to do so or that the state no longer exists in a particular region, for example, where control has been relinquished to the Islamic State. In the following section, I aim to clarify why such a duty exists, the extent of this duty, the attribution of responsibility to certain parties and whether they are overburdened by it and why it is the best solution for the problem at hand.

It is widely accepted and recognized that children who are able to flee and come to the borders of safe countries, such as those in the EU, deserve protection and that they should certainly not be returned to the conflict zones from which they fled. These legitimate claims of children are not only moral rights, but also stated in various international laws and treaties, such as the Geneva Convention Relating to the Status of Refugees, the European Charter of Fundamental Rights or case law of the European Court of Human Rights.^14^ For my case, it is not important whether this ultimately leads to granting these children refugee status or subsidiary protection. What is important is that, as soon as children arrive at a safe haven, such as within the EU, they have legitimate claims of protection from that state. One can debate how such protection has to be provided. Even such nationalists as David Miller, who denies a human right to cross borders, agree that states cannot send children, or any other person, back to a conflict zone but have to establish protection even if that is not in the country which the migrants want to enter, but in a third state (Miller 2013). So, my argument is also not concerned with the issue of prioritizing asylum seekers over other migrants^15^ but simply asserts, and this seems highly plausible, that states have a duty to ensure that they do not send children, or any other person, back to a conflict zone, where their human rights are severely endangered. Now, if we accept that those children who come to our borders are entitled to support and protection (whether or not that translates into granting asylum or transferring them to a different safe country is not of importance here), then we need to ask whether or not it would be unfair to disadvantage those children who were unable to flee and who are stuck in conflict zones. As I have shown, we cannot expect children to flee by themselves. But, if they do flee alone or even with their families, this journey is highly dangerous; it seems that it is too dangerous for this to be the basis for granting them protection. Let me use a strong analogy here: if a police officer witnesses a child being punched, held down and in immediate danger of being abused, he has the duty to intervene, not wait for that child to free himself or herself from the abuser and run towards the officer, if they are lucky enough, in order to expect any kind of child protection.

This brings me to the crucial question. So, then, even if most would agree that children’s human rights are violated in conflict zones and that states would have a duty to protect them as soon as they are at their borders, it is not so straightforward as to whether states have an obligation or duty to intervene, and even if they have an obligation to intervene, it is not clear whether this means that this intervention constitutes bringing those children to a safe haven. I will address the first issue concerning whether or not there is a duty to intervene if children’s human rights are violated. I will draw on two different sources to make my argument. The first one is Kok-Chor Tan’s argument for a duty to protect and the second is Pablo Gilabert’s argument for a positive duty to eliminate global poverty. Both are not particularly concerned with children’s human rights or the situation of children in conflict zones but, as I will show, both are applicable.

Let me begin with Tan’s argument (2006). Tan assumes rightly that an intervention is permissible in cases of severe human rights violations.^16^ The main argument against permissibility is that of state sovereignty, which is not strong enough in cases of human rights violations. The intervention I propose, as well as any intervention, would certainly be unproblematic from the perspective of state sovereignty, were the state in question to allow it and agree to allow children living in its territory to be brought to safety. Unfortunately, and for different reasons, a state like Syria may be reluctant to comply in this way, for example, because it fears that this might open up its state borders to a much more far-reaching military intervention or because it does not want to look weak in the eyes of the public or other states. Therefore, if a state refuses to let its children be brought to a safe haven, other normative arguments must be brought forward in order to legitimize such an intervention by foreign states. Firstly, as the value of state sovereignty is not universal, I would argue in particular that the respect of state sovereignty is dependent, partly at least, on the state concerned fulfilling its obligations towards its citizens by protecting their human rights. If a state fails to do that, the demand to have its sovereignty respected is weakened; in cases of gross, enduring and widespread violations, which we now witness in Syria, this argument becomes stronger. It is based both on moral and legal grounds, since the human rights of children are equally morally binding, even if they are not signed by the state in question and because most states, and Syria being one of them , have signed human rights treaties, including the CRC. So Syria has actually agreed to protect children’s rights and acknowledged that children have those rights unconditionally. Peters (2009) has argued in a similar vein that state sovereignty is not limited by human rights, but that the normative value of sovereignty is itself derived from, and oriented towards, the protection of human rights. Secondly, in the case of a violent conflict, which leads to a situation where a state actually loses control over parts of its territory and therefore cannot protect its citizens living there at all, I see it as even more legitimate for other states to intervene in the way I have proposed. Thirdly, the justification of an intervention can be strengthened if it is carried out as a joint effort by the UN, rather than by one or more foreign states. The UN does not have unlimited legitimacy to overrule state sovereignty, but it has some which is widely acknowledged and has been used in the past. Unfortunately, as the UN is dominated by interests and power games, it can lead to a situation where it cannot agree to undertake such an intervention. In this case, the duty certainly falls back on the states as a joint effort, which could lead to a situation where a coalition of states carries out its duty to bring children out of conflict zones without the backing of the UN. I would also view such an undertaking as legitimate.

So, if there are good reasons to render an intervention to protect the human rights of children as permissible, we can draw on Tan, whose aim is to show that from the permissibility that states have a duty to intervene, or to put it differently in the case of human rights violations, at least severe ones, permissibility and obligation are tied together. He argues that if human rights violations are strong enough to overrule the sovereignty of the state that is intervened with, it is also strong enough to overrule the sovereignty of the state that is obliged to intervene. Human rights violations deny neutrality and, as Tan writes in reference to Henry Shue, generate duties for all states.^17^

A second argument concerning the duty to intervene can be derived from the discussion about positive action towards the global poor based on the violations of their human rights. Gilabert (2005) has argued in opposition to Thomas Pogge, who claims that our duty towards such human rights violations are only negative ones, in favor of a positive duty to assist.^18^ Gilabert’s argument involves four steps: firstly, he wants to show that positive duties as duties of justice exist in general, such as if a person witnesses an accident and can help or that a state is obliged to tax the rich to help the poor. Secondly, he argues that we need positive duties in order to effectively eliminate poverty because it can have various causes and even if the rich (countries) stop harming the poor (countries), thus realizing their negative duties, poverty can develop and also these people have claims which can be supported. Thirdly, Gilabert distinguishes three types of robust global solidarity: (a) charity, (b) reasonable assistance to secure the conditions of autonomy and (c) harm avoidance. He holds the view that Kants’s formula of treating all persons as ends involves not only (c) but also a positive duty of (b). Fourthly, he assumes that the positive duty to assist those in poverty and to secure their human rights is not overly demanding, although some people might feel that way. He criticizes such dominant intuitions as too narrow and that they should not be taken as a benchmark for moral and political reasoning, but rather that we should criticize them.^19^ I do not see how such an argument for a positive duty to assist the poor and to protect their human rights would not be applicable to other human rights violations, such as the ones of children in conflict zones. In particular, Gilabert’s second step is interesting here. Also, in the case of the violation of human rights in conflict zones, it is unreasonable to expect that following only negative duties would be sufficient to restore the protection of human rights. As I will explore later, there is a difference in how we can and probably should assist the global poor and those in conflict zones, but in both cases positive duties to support them exist and they demand intervention.

The next crucial question is, who would be responsible for protecting the human rights of children in conflict zones, or if the best solution is to bring them to a safe haven, who is responsible for such an intervention? Tan (2006) calls this the agency question. This also implies the need to clarify the moral question about whether such a duty is a reasonable burden on those who should be held accountable. I want to propose that this duty falls to any states that are able to carry it out; not as individual states, but in terms of a joint effort, which is best assigned to the UN. This is also consistent with the pledge of the state in other human rights treaties, such as the Universal Declaration: ‘[…] Member States have pledged themselves to achieve, in co-operation with the United Nations, the promotion of universal respect for and observance of human rights and fundamental freedoms’. Let me consider some aspects of that pledge. Firstly, I am agnostic in response to questions about whether or not a global state would be the best solution to protect children’s rights, since I do not view that as a feasible or realistic option right now. Secondly, the discussion about open borders is only of limited interest to me, since I am focusing on children who have no or only very limited options to flee anyway; therefore, a regime of open borders would not benefit them very much (see, e.g. Higgins 2008). They are trapped and in need of assistance in order to get to a safe haven, where their rights can be protected effectively. Thirdly, my claim rests mainly on the assumed power of other states, and in particular their joint efforts under the heading of the UN, to effectively help those children without being overburdened by this duty. That claim certainly has two sides: the first is whether such a duty can be carried out, which I assume is possible given the significant resources that developed states have at their disposal (one argument that power provides the basis for attributing responsibility was presented in Young 2011). They have military forces, but also technical assistance and know-how in cases of natural disasters, which make it highly likely that they can effectively plan and carry out a humanitarian intervention in a foreign conflict zone. This is certainly not without its risks; but – and this is the second aspect of my claim – these risks can be limited and the protection of these children’s human rights is worth the effort. I do acknowledge that there are certain limitations to the duty I propose, which are based on what a state, or a coalition of states, can achieve, but the costs for carrying out such an intervention, as well as the subsequent protection and provision of these children, even if we must assume that this concerns hundreds of thousands of them, are most likely bearable, particularly if the burden does not fall upon one single state. Fourthly, a UN-led intervention, with the explicit goal of bringing children out of conflict zones, will limit the risk that foreign armed forces will become involved in fights within these zones. At best, a temporary ceasefire can be negotiated beforehand and the conflict parties can be involved as little or as much as is deemed necessary. The duty I propose has a clear and limited scope, which is certainly not to solve the conflict or help one side win it.

A final reason that may apply to certain states is based on the causes behind a violent conflict. Several conflicts are initiated and sustained, at least partly, by states such as the USA, Russia or Saudi Arabia because they have their own interests in a particular region. Such involvement can take many forms, ranging from supplying weapons to carrying out so-called ‘black’ operations. Based on this kind of involvement, states become liable for the consequences of their actions and making sure that children do not suffer from these consequences, or at least ensuring that their suffering is kept to a minimum (this is referred to as the liability model of responsibility in Young 2011). Since almost all states have signed and ratified the CRC or other human rights treaties, they have therefore pledged and accepted the obligation to protect children’s rights.

If we accept that children in conflict zones have a right to be protected and brought to a safe haven, this ought to also include their parents. It is already widely accepted that children have a human right to a family and identity, which covers the claim of any children to family reunification (Rohan 2015). Such a right is based on several grounds, including overcoming the trauma of separation and protecting the best interests of children. This complicates the duty I propose because it significantly enlarges the volume of those who can claim to be brought to a safe haven, as well as also involve those who are actively engaged in conflict zones and maybe do not want to leave. My answer to that problem involves two considerations: firstly, children’s rights trump parental rights (for such a child-centered view, see Archard 2004). A parent cannot decide whether a child should stay in a conflict zone; if he or she does, then he or she forfeits his or her parental rights, in which case, the state (or, in my case, foreign states and the UN) has an obligation to protect that child. Secondly, under certain circumstances, where parents would not be allowed to join their children in a safe haven, it would be justified to leave them behind. I am concerned about children’s rights in this regard and, although the right to a family is one such fundamental right, the right to be protected from getting killed or wounded, or being exploited, hungry or without shelter is more basic and should take priority.

Finally, the proposed duty to bring children to a safe haven is different from other obligations that foreign states and the UN have concerning the protection of children’s rights in general, such as violations that result from severe poverty, which is widespread in developing countries (for a detailed account on poverty and human rights, see Pogge 2011). It is certainly possible to alleviate such poverty without endangering those who are engaged in that help. Child poverty can often be effectively alleviated without bringing children to another country, although sometimes such relocation is necessary. In conflict zones, on the other hand, children need to be moved in order to escape danger and for their rights to be protected. It is possible to secure food, build sanitation and run a school in a deprived local context, but in a conflict zone, such an undertaking is almost impossible. That does not imply that I am against the far-reaching duties of rich countries to protect the rights of poor children, some of which have safely evacuated and welcomed children out of conflict zones, but this is simply beyond the scope of my paper.

## 4. Prioritizing children’s human rights

So far I have argued that an intervention to protect the human rights of children in conflict zones is permissible and obligatory and that it can attributed to the community of states and that it is an institutionalized duty, although some states may have duties based on their involvement in the conflict. But why, one can certainly ask, prioritize children’s human rights over those of others? Is it not the case that the human rights of other groups are also equally violated? What about the elderly or disabled, who are also stuck, or what about the men in torture prisons? I want to respond to these questions while drawing on two normative sources, but before that let me be clear that there exists a duty to bring children to a safe haven and this does not rule out that there is also a duty to bring other groups of people to a safe haven as well. If possible, the human rights violations need to be stopped altogether. Under non-ideal circumstances though, decisions about priorities have to be made, although I do not find it reasonable to believe that the duty I propose will use up all or even most of the resources of the rich countries. It is certainly possible to invest sufficient resources in alleviating global poverty and to bring all children out of conflict zones and protect their human rights effectively.

The first strand of arguments I want to refer to were developed by Dixon and Nussbaum (2012). They claim that children’s physical vulnerability is not sufficient basis to prioritize them, since they share this feature with other groups such as people with disability or the elderly. Instead, they say that prioritizing children’s human rights can be based on the particular vulnerability of children as socially dependent beings. They claim that the lack of control, which characterizes the children’s social position (which is both based on natural features of children and social, including legal norm), gives them a stronger claim to have their human rights protected. Dixon and Nussbaum, as I understand, argue that since being a child is a position of higher risk they deserve special protection. The second argument of Nussbaum and Dixon states that the prioritization of children’s human rights is based on a cost-effectiveness principle. They use the examples of vaccination to make their point. It is cheaper to vaccine a child then to cure her as an adult in case she gets sick. Similar reasoning, they claim, can be applied to many if not most children’s human rights.

What does that tell us about children in conflict zones? The cost-effectiveness argument has some merit and force. In Syria, hundreds of thousands of children cannot go to school and to educate them at an older age is much more costly and ineffective since children learn faster (and better under circumstances without deprivation or war). To put it in the language of the capability approach: some capabilities and functionings need to be developed early because they are the basis for others. The vulnerability principle is harder to apply to my case. In conflict zones, everyone is vulnerable and in immediate danger of getting hurt or killed or suffering other deprivations. Still I suppose this argument has some force. It seems as if the inability to protect themselves together with the knowledge that their development is easily – and often irreversibly – distorted speaks for their special treatment.^20^

A second strand of arguments that prioritize children’s human rights are widely accepted in health care policy and ethics. One is the fair innings argument (Nord 2005), which states that children’s health needs should be prioritized because they have not had their fair innings, while older people have had theirs. The fair innings argument can take two forms: either it defines a threshold, say 70 years, and discriminates everyone above that threshold because they had their fair innings, or it can be used to discriminate in cases where the age difference is substantial, say a 10-year-old and a 40-year-old. Proponents of the fair innings argument would not imply that the 40-year-old has already had her fair innings, but they would claim that she had enjoyed her life for a longer period of time than the 10-year-old meaning that the 10-year-old should be prioritized to give her the chance to also live for another 30 years. Others have advocated for a life-cycle argument, which claims that each person should have an opportunity to live through all the stages of life, because each life stage is valuable and each person should have the opportunity to experience it (Emanuel and Wertheimer 2006). While the fair innings argument looks only at the length of life, it has been argued that this is not a good measure and that we should instead look at quality adjusted life years (Ottersen 2013). If it was established that the 40-year-old can be expected to live a quality life for another 10 years, while the 10-year-old will live a quality life for only three years, we should prioritize the 40-year-old. If one also looks at the population level though, this favors children, because in general children have a greater expectancy of quality life years remaining compared to adults. As my argument covers the population of children, I suppose that the both fair innings and also the quality life years argument support my claims. It is also worth noting that the prioritization of children in healthcare has been supported from the perspective of the capability approach (Anand 2005), so it seems that Nussbaum’s and Dixon’s arguments and the fair innings and the quality of life years argument could be combined under one normative approach.

Such arguments for the prioritization of children in healthcare can be applied to the case in question, which is the violation of children’s human rights in conflict zones. Firstly, many of these violations affect children’s health directly (getting wounded, killed, or suffering from poor health due to of lack of food, medical care or sanitation). In this respect, a violent conflict is similar to the outbreak of an epidemic. Secondly, the life-cycle approach also reflects on the particularity of life stages and their value. A child that is deprived of education – which is a violation of that child’s human rights – is deprived of an important aspect of that life stage and also of a precondition to enjoy the later stages of life in a decent way. That makes the child different from the adult, who has been educated but is now not able to utilize that education. She has experienced the life stage of childhood, while the child living in the conflict zone right now has not. This argument could be expanded to say that young adults should be prioritized over older adults, but I am not exploring that further here, because my argument is focused around children.

## 5. Possible counter arguments

The first counterargument I want to discuss states that, instead of bringing children out of their home countries and into safe havens, actions ought to be concerned with improving the situation on the ground in the home countries. I do agree that this argument has some force, but I want to point out two flaws to it. Firstly, to improve the situation for children in conflict zones, such as Syria or Iraq, is a long-term project with an unclear chance of success. Children need help as soon as possible and cannot wait a few years until the situation is stabilized and healthcare and schools are back on track. Putting an end to the immediate violence is certainly a first and necessary step, but children’s rights involve more than just not getting killed by a grenade. Children, as rapidly developing beings, do not have the time to wait that long because everything they lack and miss during their childhood (e.g. nutrition, education and care) can lead to long-term effects, which are often impossible to be dealt with later. Furthermore, it is not foreseeable whether such peace-building will work altogether or whether it will fail, thereby making the situation even worse for children. As long as it remains plausible that children’s rights will actually be reinstated and protected in the process of bringing children out of conflict zone, we should not gamble with children’s lives in that way. Secondly, the improvement of children’s rights in their home country will certainly be more demanding than the duty to bring them to a safe haven. History tells us that such peace-building, followed by nation-building, is a high-cost, high-risk adventure with uncertain results. It would probably involve much more engagement in those states than the humanitarian missions that are linked to my proposal. Bringing children out of conflict zones is also dangerous and will also probably involve on-the-ground operations, but they will be on a much smaller scale than those required for deciding the conflict and rebuilding a functioning state, both of which can guarantee children’s rights. Furthermore, as I have said, evacuating children does not interfere with the conflict itself, so those engaged in the conflict have fewer reasons to oppose it or even fight those who are getting the children out. The situation is completely different when it comes to peace- and nation-building, which almost certainly implies that there will be one side that has to lose who will fight those who help the winning side. Such a duty is, therefore, linked to a much higher risk and would demand much more involvement on the side of duty-bearing states. Instead of saying that such a duty may be justified, it seems obvious to me that, from a children’s rights perspective, the solution that is more effective and has a better chance of successfully protecting children’s rights should be prioritized, which is the duty to bring children to safety first and foremost. That said, my argument does not necessarily imply that all children from conflict zones need to be brought to the EU or the USA. The duty I am arguing for demands to bring them to a safe haven, where their rights are sufficiently protected. That can be in a safe zone within the country or in a neighboring country. Only if there are good reasons to believe and sufficient evidence that their rights can only be protected by bringing them to the EU or to any other highly developed country should this be the solution.

A second counterargument could be that those states, which have such a duty to bring children to a safe haven, would be overburdened by it. Is it feasible to assume that states can take in millions of children (and their families)? Without hoping to do justice to the extensive literature on the issue of overdemandingness (see e.g., Sonderholm 2013) I only want to make one point in that respect. Firstly, it seems plausible to assume that, as a collective, the highly developed states which share this duty that I propose would be able to take in and to take care of millions of children (and their families). The crucial question here is how much can we demand from them. Following a Singerian account (Singer 2010), for example, one could assert that as long as the loss in welfare in those countries, which take in these children, does not push them below a sufficient limit of basic welfare, they have an obligation to do so. A more convincing account, in my opinion, would assert that highly developed countries have a duty to sufficiently protect the rights of children (and their families) as well as other people with equal claims as long as it does not endanger the protection of the rights of their citizens, which includes a certain level of welfare and also demands that the state does not deplete all or most of its resources to protect children in conflict zones. The question of when this threshold is reached, so if it is possible to bring 2 million or 20 million children to a safe haven, or if the EU can take in 5 or 25 million refugees before being overburdened, is an empirical one that I cannot answer here.^21^ For example, the German Government calculated it will spend about 100 Billion Euros in total for the two million refugees they expect until 2020.^22^ A large sum but still only a small fraction of its annual GDP of 3.3 trillion. Certainly the costs associated with the duty to bring children to a safe haven would be lower if these children would be able to live in safe zones in the region and could be provided with everything they need there.^23^ The solution I propose is certainly limited by such external factors, as I also acknowledged throughout this paper, but this does not mean that no such solution exists in the first place, and that the highly developed countries are not able to fulfill it properly. At least from an economic point of view, they have more than enough resources to take care of millions of children.

The third, and final, counterargument I want to discuss is whether such a duty would also demand a military intervention, which could worsen the situation even further (for a detailed discussion of potential dangers, see Kydd and Straus 2013). I concede that this danger exists, but it may be limited. The first limiting factor depends upon whom is carrying out the intervention. If it is done by the UN, as I suggest, it is clear that a neutral force would be intervening with a limited agenda. That makes it more likely that these forces, which are necessary to carry out the intervention, will not be targeted or become involved in the conflict. Even if the intervention is carried out by a coalition of foreign states, however, the risks of being dragged into the conflict may be limited, even to a great extent. It needs to be made clear that such an intervention is not being used as a pretense to support one side over the other or to achieve other goals, such as securing access to natural resources. Secondly, the intervention needs to be well-coordinated and carried out without delaying the process. If hundreds of thousands or even millions of children are affected, it will certainly take some time to reach those trapped in conflict zones, where actual war is going on, which comes with certain risks and will certainly not be easy. In such cases, negotiating with the parties in conflict will most likely be the best approach, for example, in order to reach a temporary ceasefire during which those children can be evacuated. It might even be the case that risks to the well-being of those who have sent in to carry out the intervention are too high compared to the possible gains. Such decisions can only be made on a case-by-case basis and, although I wish I were able to make a strong argument for the solution I propose, I am aware that other arguments may outweigh it under certain circumstances. States certainly also have a duty to work towards peace, in particular those which are actively involved in the ongoing war, and to resolve the situation for the better. In the meantime, though, those children who are suffering from the conflict should be brought to a safe haven, where they have their rights sufficiently protected and can wait in peace for the end of the conflict, which made it impossible for them to live there without harm or danger to their life and well-being.

## 6. Conclusions

I want to conclude my paper with some thoughts on borderline cases, which are actually very important in relation to what is actually happening today. Many children have already left the conflict zones in Syria and moved to safer places there or in neighboring countries, such as Turkey, Lebanon or Jordan. What about these hundreds of thousands of children? Does my duty to bring children to safety also cover them? I would like to say that an expansion of my argument also seems plausible in order to include those children because it is very likely that some of their rights are also being grossly violated. Living in a refugee camp comes with many hardships and restrictions, for example, the lack of adequate nutrition or access to healthcare, education and shelter. Children under such circumstances are often not in immediate danger of dying, but there are convincing arguments that they have justified claims to a better quality of life under the framework of the CRC. These children are also more or less stuck where they are and have little or no chance to improve their situation, at least not on their own, while attempting to move to a European country, such as Austria or Germany, is highly dangerous. It should also not be forgotten that being in a safer zone in Syria or Iraq right now does not necessarily mean that the conflict stays where it is. Even if I am convinced, however, that children in such refugee camps in neighboring countries or living in safer locations in Syria have claims to be brought to safety, I still think that we should prioritize those children who are stuck in conflict zones. The immediate danger for children is greater there.

## Notes

1. See the text of the CRC: http://www.ohchr.org/en/professionalinterest/pages/crc.aspx.

2. I am assuming children’s rights here as both legal and moral rights for two reasons: firstly, the CRC is a legal document but one which also articulates moral rights and is based, so I assume, also on moral grounds. Secondly, I propose a solution that is primarily a political one – a duty of states and the international community of states – and for that legal considerations on the human rights level are of utmost importance. That said, I am well aware that I am arguing mostly from a moral point of view, but one that hopefully can be connected to the legal dimension of children’s rights.

3. Also this claim seem uncontroversial, although there is considerable controversy what follows from the universality of human rights. An argument for the universality of human rights as the necessary content of global justice has been brought forward by David Miller, who is skeptical of the far-reaching claims of cosmpolitanism (Miller 2008).

4. UNICEF has set up and a webpage about the situation of children in Syria, where the newest information can be found: http://childrenofsyria.info.

5. 
http://www.un.org/apps/news/story.asp?NewsID=53145.

6. 
http://reliefweb.int/report/syrian-arab-republic/unicef-syria-country-office-nutrition-facts-figures-september-2015.

7. 
http://childrenofsyria.info/2016/03/31/syria-all-children-everywhere-now-urgent-funding-needs/.

8. 
https://www.worldvision.org/wv/news/Syria-war-refugee-crisis-FAQ.

9. 
http://www.aljazeera.com/news/2015/07/syrian-children-increasingly-exploited-labour-150702061339627.html.

10. This reflects the problem that not all children are more vulnerable than all adults, for example chronically sick or disabled adults, but that taken as a group children are more vulnerable than the group of adults. More about the complexity of children’s vulnerability can be read here Macleod (2015).

11. The CRC is very clear that, for younger children, this is also a clear violation of children’s rights: ‘States Parties shall take all feasible measures to ensure that persons who have not attained the age of fifteen years do not take a direct part in hostilities’ (§38). I would assume that the CRC is not strict enough in this regard and that children should not be recruited until the age of 18 years, but this lies beyond the scope of my argument here.

12. The case of child soldiers shows how complicated things can get here. Children who are recruited to fight are certainly victims, but they are also not free of all responsibility for their actions. For some child soldiers, returning to a normal childhood is also difficult, maybe even impossible, because they do not want to be treated as children anymore, as they feel and behave adult-like. See, for example, Özerdem and Podder (2011).

13. Such an analysis of the competences of a child, for example, to decide where to live and with whom, is common in custody cases in some countries. To this extent, I would also assume that children should not be allowed to decide whether to stay in a conflict zone. Their view on this matter is not authoritative. For more discussion on this topic, see Archard and Skivenes (2009).

14. See the legal report, ‘European Union: Status of Unaccompanied Children Arriving at the EU Borders’: https://www.loc.gov/law/help/unaccompanied-children/eu.php.

15. There are good reasons – political ones mainly – to treat migrants differently according to whether they are asylum seekers or not but that does not affect my argument. See: Carens (1992).

16. The literature on these issues is vast, and it is not the aim of this article to add something new to the general debate about humanitarian interventions to protect human rights but to propose and discuss a specific duty that rests on the permissibility of humanitarian interventions, or at least some of them. For an overview see (Hehir 2013).

17. According to Shue (1996) human rights involve three types of duties: the duty to avoid human rights violations, the duty to protect people from human rights violations and the duty to assist people, whose human rights have been violated. In the case of a conflict zone, the respective state fails to do all three, and thus an external agent needs to step in.

18. Gilabert is certainly not the first to argue for such a positive duty. Another theorist would be David Miller, who is in general, as said, skeptical toward duties of global justice, but also he states that remedial duties exist in cases of human rights violations, for example a dictator that deprives his people (Miller 2007, 231–259). But, again, I do not aim to add much to the basics of the debate about positive or negative duties but want to show that if a positive duty to protect the human rights of children exists that this should take the form of such a duty to bring them to a safe haven in cases that they are trapped in conflict zones.

19. A more detailed criticism of the ‘overdemandingness’ objection against positive duties has been elaborated by Sonderholm (2013).

20. Colin Macleod has made a similar argument, some while ago. He writes (Macleod 2002, 224):
First, meeting children’s claims to just treatment seems to enjoy a general priority over meeting the comparable claims of adults. Let me try to motivate this claim through an example. There is a sense in which children and adults have a similar interest in avoiding suffering severe pain that can give rise to a comparably strong entitlement to access to pain medication. Yet where circumstances force a choice between providing pain-relieving medication to a child and providing medication to adult suffering the same pain, we seem to have reason to give priority to recognition of the child’s claim. I suspect that this general priority is grounded in various related factors. Children are vulnerable and dependent in many ways on adults for protection of their most basic interests. Because they cannot effectively represent and secure their own interests we naturally attach moral urgency to ensuring they receive fair treatment. The fact that children are developmentally fragile also seems significant. Children often seem to suffer more and are less able to recover from the ill effects of unjust treatment. So even when their moral claims seem comparable, caution suggests favouring the claims of children. More generally, children’s status as innocents who can be assigned no responsibility for their plight or for ameliorating unjust treatment they face supports a general priority of children’s claims. There seems to be an important difference in the relative urgency of the particular competing entitlements in this case. In sum, moral ties go to children.

21. Research suggests, contrary to the public opinion and some voices in politics, that the economic burden of the migration movement to the EU in 2015 is in fact low. The situation for children is certainly a bit different but in the long-run similar positive effects can be expected. One recent economic simulation by the Economics and Econometrics Research Institute concludes:
Our simulation results suggest that, although the refugee integration (e.g. by providing welfare benefits, language and professional training) is costly for public finances, in the medium – to long – run the socio-economic and fiscal benefits significantly outweigh the associated refugee integration costs. (Kancs and Lecca 2016, 26)

22. 
http://www.reuters.com/article/us-europe-migrants-germany-costs-idUSKCN0Y50DY.

23. A newspaper article in the *The Independent* calculates the costs for one refugee to be 10 times lower in a camp in Jordan. http://www.independent.co.uk/voices/syrian-refugees-will-cost-ten-times-more-to-care-for-in-europe-than-in-neighboring-countries-a6928676.html.

## References

[CIT0001] Anand Paul (2005). Capabilities and Health. *Journal of Medical Ethics*.

[CIT0002] Anderson Joel, Claassen Rutger (2012). Sailing Alone: Teenage Autonomy and Regimes of Childhood. *Law and Philosophy*.

[CIT0003] Archard David (2004). *Children: Rights and Childhood*.

[CIT0004] Archard David, Skivenes Marit (2009). Balancing a Child’s Best Interests and a Child’s Views. *The International Journal of Children’s Rights*.

[CIT0005] Berman Helene (2001). Children and War: Current Understandings and Future Directions. *Public Health Nursing*.

[CIT0006] Carens Joseph H. (1992). Refugees and the Limits of Obligation. *Public Affairs Quarterly*.

[CIT0007] Devakumar Delan, Birch Marion, Rubenstein Leonard S., Osrin David, Sondorp Egbert, Wells Jonathan C. K. (2015). Child Health in Syria: Recognising the Lasting Effects of Warfare on Health. *Conflict and Health*.

[CIT0008] Dixon Rosalind, Nussbaum Martha (2012). Children’s Rights and a Capabilities Approach: The Question of Special Priority. *Cornell Law Review*.

[CIT0009] Emanuel Ezekiel J., Wertheimer Alan (2006). Who Should Get Influenza Vaccine When Not All Can?. *Science*.

[CIT0010] Gilabert Pablo (2005). The Duty to Eradicate Global Poverty: Positive or Negative?. *Ethical Theory and Moral Practice*.

[CIT0011] Hehir Aidan (2013). *Humanitarian Intervention: An Introduction*.

[CIT0012] Higgins Peter (2008). Open Borders and the Right to Immigration. *Human Rights Review*.

[CIT0013] Kancs d’Artis, Lecca Patrizio (2016). http://www.eeri.eu/documents/wp/EERI_RP_2016_08.pdf.

[CIT0014] Kanics Jyothi, Senovilla Hernández Daniel, Touzenis Kristina, Unesco (2010). *Migrating Alone: Unaccompanied and Separated Children’s Migration to Europe*.

[CIT0015] Kydd Andrew H., Straus Scott (2013). The Road to Hell? Third-Party Intervention to Prevent Atrocities: THE ROAD TO HELL?. *American Journal of Political Science*.

[CIT0016] Macleod Colin, Archard David, Macleod Colin (2002). Liberal Equality and the Affective Family. *The Moral and Political Status of Children*.

[CIT0017] Macleod Colin, Bagattini Alexander, Macleod Colin (2015). Agency, Authority and the Vulnerability of Children. *The Nature of Children’s Well-Being*.

[CIT0018] Miller David (2007). *National Responsibility and Global Justice*.

[CIT0019] Miller David (2008). National Responsibility and Global Justice. *Critical Review of International Social and Political Philosophy*.

[CIT0020] Miller David (2013). Border Regimes and Human Rights. *The Law & Ethics of Human Rights*.

[CIT0021] Nord Erik (2005). Concerns for the Worse off: Fair Innings Versus Severity. *Social Science & Medicine*.

[CIT0022] Ottersen Trygve (2013). Lifetime QALY Prioritarianism in Priority Setting. *Journal of Medical Ethics*.

[CIT0023] Özerdem Alpaslan, Podder Sukanya (2011). *Child Soldiers: From Recruitment to Reintegration*.

[CIT0024] Peters Anne (2009). Humanity as the A and Ω of Sovereignty. *European Journal of International Law*.

[CIT0025] Pogge Thomas (2011). Allowing the Poor to Share the Earth. *Journal of Moral Philosophy*.

[CIT0026] Quosh Constanze, Eloul Liyam, Ajlani Rawan (2013). Mental Health of Refugees and Displaced Persons in Syria and Surrounding Countries: A Systematic Review. *Intervention*.

[CIT0027] Rohan Mark (2015). Refugee Family Reunification Rights: A Basis in the European Court of Human Rights’ Family Reunification Jurisprudence. *Chicago Journal of International Law*.

[CIT0028] Schapiro Tamar (1999). What Is a Child?. *Ethics*.

[CIT0029] Shue Henry (1996). *Basic Rights. Subsistence, Affluence, and U.S. Foreign Policy*.

[CIT0030] Singer Peter (2010). *The Life You Can Save: How to Play Your Part in Ending World Poverty*.

[CIT0031] Sonderholm Jorn (2013). World Poverty, Positive Duties, and the Overdemandingness Objection. *Politics, Philosophy & Economics*.

[CIT0032] Tan Kok-Chor (2006). The Duty to Protect. *Nomos*.

[CIT0033] Young Iris Marion (2011). *Responsibility for Justice*.

